# Pleomorphic Xanthoastrocytoma; Clinicopathological spectrum of An Intriguing neoplasm

**DOI:** 10.12669/pjms.342.14663

**Published:** 2018

**Authors:** Mariam Abid, Saroona Haroon, Aisha Hassan Memon, Zubair Ahmad, Sheema Habib Hasan

**Affiliations:** 1 Dr. Mariam Abid, FCPS. Department of Pathology, Shifa College of Medicine, Shifa Tameer-e-Millat University, Islamabad, Pakistan; 2 Dr. Saroona Haroon, FCPS. Prince Faisal Cancer Centre, King Fahad Specialist Hospital, Buraidah, Kingdom of Saudi Arabia; 3 Dr. Aisha Memon, FCPS. Histopathology Section, Department of Pathology and Microbiology, Aga Khan University Hospital, Karachi, Pakistan; 4 Dr. Zubair Ahmad, FCPS. Histopathology Section, Department of Pathology and Microbiology, Aga Khan University Hospital, Karachi, Pakistan; 5 Prof. Sheema H Hasan, FRC-PATH. Histopathology Section, Department of Pathology and Microbiology, Aga Khan University Hospital, Karachi, Pakistan

**Keywords:** CNS tumor, Pleomorphic xanthoastrocytoma, Astrocytoma, Gliomas

## Abstract

**Background & Objective:**

Pleomorphic xanthoastrocytoma (PXA) is a rare primary WHO Grade II astrocytic tumor comprising of < 1% of all astrocytomas. It is generally benign and slow growing however disease progression and malignant transformation with anaplastic features have been infrequently reported. Our objective was to assess clinicopathological characteristics of this rare tumor at our center.

**Methods:**

A retrospective study was conducted at Aga Khan University Hospital from January 1992 till January 2016. Data was entered on a proforma including patient demographics, clinical features, tumor location, histological features and follow-up, where available.

**Results:**

Forty Seven cases of PXA were retrieved during the study period. The mean age was 23.8 years (SD=15.1) and median age was 19 years. The most frequent symptom was head ache (n=31). Male were more frequently affected (n=26). The commonest location was temporal lobe. On microscopic examination, tumors were pleomorphic without mitoses or necrosis, however two cases showed increased mitotic activity, and one case revealed associated gliosarcoma. Follow-up of only 29 cases was available for a period ranging between 2 and 184 months (85 months +/- 56 months). Outcome was good in 27 patients with the last follow up showing no radiographic or clinical evidence of tumor recurrence.

**Conclusions:**

PXA is an infrequent tumor in our population also, with less than 50 cases identified in two decades study period. Due to its rarity and its bizarre histomorphology, it should be diagnosed correctly, as it has got better prognosis than other astrocytic tumors.

## INTRODUCTION

Pleomorphic xanthoastrocytoma (PXA) which was originally described in detail by Kepes et al. is an uncommon tumor that comprises of approximately 1% of all astrocytic central nervous system (CNS) tumors.^1^-^3^ The most common presentation is seizure. Most of the PXAs are located supratentorially. Despite the ominous histologic features as marked cellular pleomorphism and lipidization, PXAs have a favorable prognosis and are classified as Grade II in the World Health Organization (WHO) classification.^4^,^5^ However few histologic features such as necrosis, significant nuclear atypia and many mitoses are indicative of a high grade tumor. The ideal is gross total resection, which offers an excellent prognosis.^6^-^8^

Not a single comprehensive study has been done in Pakistan, which describes in detail, clinical and pathological characteristics of this tumor. In this study, we describe the clinicopathologic features and prognostic factors of 47 patients with PXAs in Southern Pakistani population.

## METHODS

We reviewed the histopathology reports with pertinent information along with the slides of surgical specimens of all patients diagnosed as PXA in Histopathology department, Aga Khan University hospital from January 1992 till January 2016 with total period of 24 years.

Forty Seven consecutive patients were diagnosed with PXA based on the pathologic features described in the WHO classification of tumors of the central nervous system (2007). The PXAs that demonstrated increased mitotic activity (defined as 5 or more mitoses per 10 high-power fields) with or without areas of necrosis were designated as “PXA with anaplastic features” according to WHO classification.

The slides were reviewed and presence of necrosis, mitotic figures and Ki-67 were carefully evaluated by two pathologists (each having experience of more than three years post-fellowship). A comprehensive analysis was done on these patients regarding clinical and pathologic features at time of diagnosis as well as treatment and follow-up information, where available.

All the details were recorded on a Performa. Percentages were calculated for the frequencies of numerical data. Four cases in which only reports were available and slides could not be obtained for review, from the record, were also excluded from the study. The cases reported as “astrocytoma with PXA-like features” were not included in the study.

**Fig.1:**
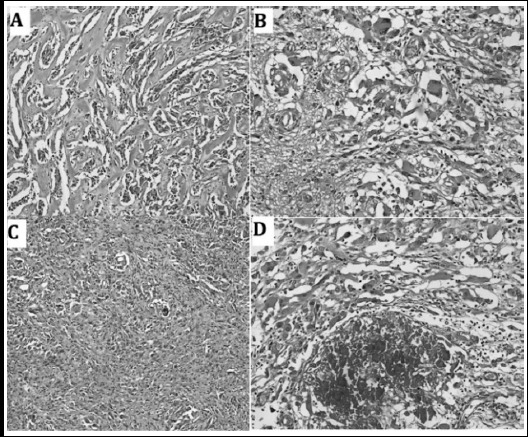
Hematoxylin and Eosin stained sections at medium power show pleomorphic cells with giant cells (A&B). Spindling of the tumor cells (C) and Calcification (D) can also be seen.

Departmental ethical clearance was obtained from the Department of Histopathology, AKUH. Institutional Ethical board review approval was not sought for, as no patient identifiable material was used in the study.

## RESULTS

Total 47 cases were retrieved from archives of department of histopathology, from January 1992 to January 2016 during period of 24 years. Male:female ratio was 1.9: 1 and mean age was 24.2 years. Median age was 19 and range was 3 – 62 years. Apart from few patients, majority of the patients presented with tumors in the supratentorial hemispheres. In our study the most common location was temporal lobe followed by parietal lobe and frontal lobe. Other sites included lateral ventricles (n=3), cerebellum (n=2) and spinal cord (1 patient). Symptoms at presentation were seizures only (n=28) and seizures with neurologic deficits (n=11) and headache only (n=8). Prominent fascicular pattern of the spindle cells was seen in most of the tumors but two tumors revealed spindle cells in haphazard pattern. Eosinophilic granular bodies (EGB) were also found frequently (n=45). Mitotic counts ranged from 0 to 9/10 HPFs. five tumors contained mitoses above 5/10HPF and all of these showed necrosis also. Microvascular proliferation was present in two of these 5 cases. All of these five cases were diagnosed as PXA with Anaplastic features. Perivascular lymphocyte cuffing was identified in 40 cases (85%). One case showed malignant mesenchymal component also with diagnosis of Gliosarcoma with PXA. The Ki-67 labeling index was >5% in five tumors. The tumor cells were positive for GFAP in all cases. S-100 protein and Synaptophysin were present in 75% of tumors, with a mean MIB-1/Ki-67 proliferative labeling index of 6%. Clinical features have been briefly outlined in Table-I.

**Table-I:** Salient Clinical features. (n=47).

Characteristics	No. of cases (Percentage)
*Age*	
<20 years	26 (55.3%)
>20 years	21 (44.7%)
*Gender:*	
Male	32 (68.1%)
Female	15 (31.9%)
*Location:*	
Supratentorial	44 (93.6%)
Infratentorial	3 (6.4%)
Presenting symptoms	
Seizures only	28 (59.6)
Headache only	8 (17%)
>1 symptoms, including seizures	11 (23.4%)

**Fig.2:**
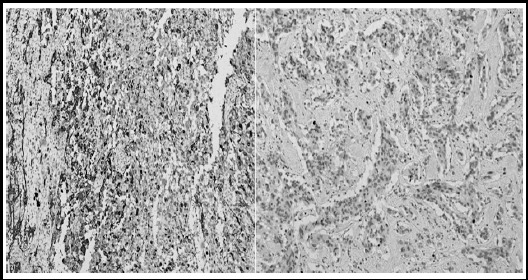
Immunohistochemical stain Glial Fibrillary Acidic Protein [GFAP] (A) and Ki-67 Proliferation index [MIB-1] (B).

Follow-up of only 29 cases was available for a period ranging between 2 and 184 months (85 months +/- 56 months). Outcome was good in 25 patients with the last follow up showing no radiographic or clinical evidence of tumor recurrence. Three patients died following progression of the disease; one at three months and two had recurrence at 15 and 23 months respectively. One more patient showed recurrence but was treated successfully.

## DISCUSSION

Gliomas are the largest group of primary central nervous system (CNS) tumors and comprise of variety of clinically and genetically distinct subclasses. Diffuse gliomas, which include astrocytomas, oligodendrogliomas and oligoastrocytomas are the most common type and are characterized by their infiltrative nature.^1^,^9^ They occur predominantly in adults. Pilocytic astrocytomas (WHO Grade I) and Pleomorphic Xanthoastrocytomas (PXA) (WHO Grade II) are pediatric neoplasms which are relatively circumscribed, offering the possibility of cure following complete resection.^10^-^13^

Pleomorphic xanthoastrocytoma (PXA) is rare primary neoplasm of brain. Although histopathologic features suggest high grade tumor, PXA is a low-grade gliomas corresponding to WHO Grade II and in spite of its pleomorphic appearance, it has a relatively good prognosis. It was added in the 1993 World Health Organization (WHO) classification of tumors of the CNS.^14^ Although PXA is an uncommon tumor, it has enthralled researchers worldwide. Many studies have been published since the description of the tumor. However most of the literature is from the West and very few cases from the Asian subcontinent and even fewer have been reported from Pakistan.^13^,^14^

To date, this is the largest study to encompass the morphological details and frequency of PXA in our population in Southern Pakistan. As per Ahmed Z et al., 1110 cases of CNS neoplasms were reported over a six year period however, their recent data showed that 597 cases were reported in the year 2008 alone. So the overall incidence has been on increase in the region. They reported PXA comprising 2.8 % of all CNS glial tumors.^15^

Advances in the knowledge of the natural history of PXA and itsprognosis have been hampered by its rarity. PXA has a relatively favorable prognosis than other infiltrative astrocytomas, with 30% recurrence rate and 75% to 80% overall survival rates following primary resection at 5 years of follow-up.^5^,^7^

Pleomorphic Xanthoastrocytoma (PXA) is mostly seen in children and young adults.^8^,^16^ We also observed it in majority of children, however 10 patients more than 40 years of age. The oldest patient with PXA was 62 years. Primary pleomorphic xanthoastrocytoma (PXA) of the spinal cord is a very rare slow growing tumor and very few cases have been reported to date. We encountered one spinal PXA also.

Histologically, PXA is characterized by spindle cells, multinucleated giant cells and foamy lipid-laden xanthomatous cells. Mitosis and necrosis is commonly absent.^17^,^18^ Although PXA usually appears as a highly cellular astrocytic tumor composed of large, bipolar cells having pleomorphic appearance and spindled appearance with mononucleated and multinucleated giant cells. The nuclei are dark and lobulated and there is vacuolated glassy cytoplasm with the eosinophilic granular cell bodies and reticulin fibers in the background. The dense cellularity and pleomorphism raises a possibility of ganglioglioma and higher grade malignancies like glioblastoma, giant cell glioblastoma, gliosarcoma or even pleomorphic sarcoma. However necrosis is usually absent and mitoses are low. Few PXA can show increased mitoses with >5 / 10 High Power Field (HPF) along with necrosis which are termed as PXA with Anaplastic features. Less than one third of the PXA shows anaplastic features and this has a bad prognosis. In our study, we found 5 (10%) anaplastic PXA, much less than 31% found in study by Ida CM et al.^2^

This tumor develops chiefly at superficial cortical regions with or without partial involvement of leptomeninges and in the supracortical location, it occurs more preferably within the temporal lobes.^19^-^21^ We had significant number of tumor seen in 21 patients in temporal lobes, followed by parietal and frontal lobes. Interestingly intraventricular location was observed in three cases, which is again an infrequent site for this neoplasm. No multifocal or multiple tumors were identified.

Immunohistochemical studies demonstrated diffuse positivity for Glial Fibrillary Acidic Protein (GFAP) and Vimentin in all the tumors. In literature it has been reported that about 70% of cases show Immunostain CD34 positivity. We also saw CD34 positivity in 20 (76.9%) out of 26 cases, in which it was applied.

PXA associated with other tumors as collision tumors have also been rarely described. We received one case of PXA with gliosarcoma or we can word it as Gliosarcoma arising in PXA. Approximately 40% of PXA are reported to recur within 10 years of primary resection. Upon recurrence, patients receive radiation therapy and conventional chemotherapeutics designed for high-grade gliomas. Genetic changes, that can be targeted in the recurrent tumors, for the treatment purposes. In our study three cases recurred but the recurrence factors could not be assessed in detail as surgical extent of resection details were not available. All the three cases were having Anaplastic features in the initial diagnosis. Giannini et al indicated the mitotic index as the most significant predictor of recurrence and survival in a multivariate analysis.^3^ They suggested that the mitotic index >5 should be referred to as PXA with anaplastic features, as it denotes poor outcome group.^20^,^22^ Our findings also support this suggestion as all the recurrent cases had mitoses more than 5 / 10 HPF.

Ki67 proliferation index is also usually very low and in our study, Ki67 was applied on 38 cases and out of these, five cases showed Ki67 >5%. The highest Ki67 proliferation was 20%, a case with anaplastic features, which also showed early recurrence. Treatment details were deficient apart from the recurrent cases, 2 of those had GTR and one had chemotherapy.

## CONCLUSION

PXA is rare tumor and we observed 1.6% PXAs of all Astrocytic tumors in Southern Pakistani population. In our population, it is not limited to pediatric population only and it can be diagnosed in adult population as well. Keeping in view, its good prognosis and different course of progression from other high grade gliomas, this should be entertained in the differential diagnosis of morphologically high grade entities by pathologists.

### Authors’ Contribution

**MA, SH and AHM** conceived, designed and did statistical analysis & editing of manuscript.

**SHH, MA, SH & ZA** did data collection and manuscript writing. SH and MA did review and final approval of manuscript.
